# Reoperation rates for recurrence of fibroids after abdominal myomectomy in women with large uterus

**DOI:** 10.1371/journal.pone.0261085

**Published:** 2021-12-09

**Authors:** Katherine J. Kramer, Sarah Ottum, Damla Gonullu, Capricia Bell, Hanna Ozbeki, Jay M. Berman, Maurice-Andre Recanati

**Affiliations:** 1 Department of Obstetrics and Gynecology, St. Vincent’s Medical Centers Manhattan, New York, New York, United States of America; 2 Department of Obstetrics and Gynecology, University of Cincinnati, Cincinnati, Ohio, United States of America; 3 Department of Obstetrics and Gynecology, Wayne State University School of Medicine, Detroit, Michigan, United States of America; 4 Wayne State University School of Medicine, Detroit, Michigan, United States of America; 5 Milken Institute of Public Health, George Washington University, Washington, DC, United States of America; 6 NIH-Women’s Reproductive Health Research (WRHR) Scholar, Department of Obstetrics and Gynecology, Wayne State University, Detroit, Michigan, United States of America; Ohio State University Wexner Medical Center Department of Surgery, UNITED STATES

## Abstract

**Background:**

The population of women undergoing abdominal myomectomy for symptomatic large fibroid uterus is unique. We seek to characterize the timing, risk factors as well as the presenting symptoms which led patients to undergo repeat surgery in this patient population.

**Methods and findings:**

We followed 592 patients who underwent an abdominal myomectomy from March 1998 to June 2010 at St. Vincent’s Catholic Medical Center and presented later during the study period with a recurrence of symptoms attributable to a reemergence of fibroids and who chose to undergo repeat surgical management. Twelve percent of patients exhibited symptoms of fibroid uterus which led to reoperation within the study period. The mean age at repeat surgery was 44.1 ± 0.6 years old (n = 69) and the mean time between operations was 7.9 ± 0.3 years. Presentation was variable but included bleeding, pain and infertility. Patients presented for surgery with a significantly smaller sized uterus than at their initial surgery. Timing between surgeries correlated with age at initial surgery and uterine size but race, number of fibroids, aggregate weight of fibroids removed, operative time or blood loss at the initial surgery did not correlate. Data is suggestive that intraperitoneal triamcinolone may reduce reoperation rates but not timing of recurrence.

**Conclusion:**

These results may help in counseling patients, particularly younger women, on the risks of fibroid recurrence necessitating repeat surgery. Further research is necessary to assess if triamcinolone can alter fibroid reurrence in patients who undergo uterus sparing procedures.

## 1. Introduction

Uterine fibroids, or leiomyomas, are the most common benign tumors in premenopausal women. They are usually discovered incidentally by imaging in asymptomatic women, but 20%-50% of women develop symptoms such as abnormal uterine bleeding, pelvic pressure, pain or urinary or bowel complaints [1]. Some risk factors for the formation of these benign tumors include: Black race, age, family history, premenopausal state and hypertension, while use of hormonal contraception, smoking in low BMI women and low parity are protective [2]. Black women have the highest lifetime risk of having fibroids and suffer from more severe symptoms, which interfere with daily functioning [3].

Non-surgical treatment of uterine fibroids include expectant management [4] and medical therapy, which include estrogen-progestin contraceptives, progestins [5, 6], levonorgesterel releasing intrauterine devices [7] and implants, progesterone receptor modulators [8] such as ulipristal acetate [9], mifepristone [10], and GnRH agonists [11], aromatase inhibitors [12] and selective estrogen receptor modulators such as raloxifene [13]. These approaches may provide symptom relief, especially in situations where bleeding is the main complaint. Although about three quarters of women report short-term improvement over the first year of treatment, long-term failure rates are high [14], and about 50% will have surgery within 24 months [15]. The surgical approach is the principal treatment of fibroids, especially in cases where bulk-symptoms are the dominant issue or if infertility is attributable to the presence of myomas. Hysterectomy as well as modern surgical approaches such as uterine artery embolization, radio frequency ablation and magnetic resonance-guided focused ultrasound can provide a cure for symptoms. Myomectomy, which may be performed laparoscopically or hysteroscopically, is recommended for women, who wish to maintain fertility. With any uterine conserving approach, however, the risk of fibroid recurrence remains.

Around 15–33% of fibroids recur after myomectomy, and around 10%-21% of women undergo a hysterectomy within five to ten years [16, 17]. Published rates and time to recurrence vary widely and include 12–15%, 31–43%, 51–62%, and 84% at 1, 3, 5, and 8 years respectively [18–22]. In this study, we focus on a group of 64 women, who were admitted for uterine surgery after having previously undergone an open myomectomy for a large symptomatic fibroid uterus with a mean size equivalent to 20.9 ±0.5 weeks gestation (range: 12–30 week-size). While the recurrence rates published in the literature address recurrence of fibroids in general [23] or the rates of reoperation following myomectomy [17], our study specifically addresses reoperation rates in patients, who have undergone myomectomy using an open approach for an initial diagnosis of large symptomatic fibroid uterus. The current literature also does not typically address the reasons underlying the need for repeat surgery. We seek to characterize the timing, risk factors as well as the presenting symptoms which led patients to undergo repeat surgery in this patient population.

## 2. Materials and methods

### 2.1 Patient selection

Admissions for uterine surgery after previous myomectomy from March 1, 1998 to June 2010 were identified by the St. Vincent’s Catholic Medical Center Manhattan medical records office and through office charts under IRB approval (#0104191MX). A subset of this deidentified dataset was previously queried for a separate unrelated study evaluating postoperative adhesions. Tabulated fields included, data from the initial surgery (age, uterine size, number and aggregate weight of fibroids removed, surgical time and estimated blood loss and initial surgeon name). Uterus size was determined clinically via bimanual exam performed by the attending surgeon and described in menstrual weeks as with the gravid uterus as well as by abdominal and transvaginal ultrasound. All women were continuously enrolled and followed during the study period (range: 1 to 12 years). Fields from the office visit when the surgeon evaluated the patient with new complaints included: symptoms, alternate treatments received thus far as well as uterine size. Patients were evaluated preoperatively with pelvic exam, transvaginal ultrasound, endometrial biopsy, liquid-based cervical smear, complete blood count, type and screen, coagulation studies, thyroid function tests, follicular stimulating hormone and serum human chorionic gonadotropin (hCG). Chart review documented that patients were appropriately counselled on alternative approaches including watchful waiting, medical management and minimally invasive surgical approaches including repeat myomectomy and hysterectomy. Data collected from the electronic hospital chart at reoperation included age, uterine size, surgical complications and name of surgeon performing the procedure. Total number of myomectomies performed as well as total number which involved the instillation of the intraperitoneal steroid triamcinolone, during a previous study at the institution, were obtained from medical records.

### 2.2 Surgical methods

We recorded, from the operative note, if any anti-adhesive methods were employed at all at the initial surgery or if the patient received no such treatment. This decision is left to the surgeon at our institution. Such methods potentially included either the use of adhesion barriers: principally at our institution Interceed (Johnson&Johnson, New Brunswick, NJ) or Seprafilm (Baxter, Deerfield, IL); or the instillation of intraperitoneal steroids. This latter method consisted of intraperitoneal placement of 200 mg triamcinolone acetonide suspension (Bristol-Myers-Squibb Pharmaceutical, Princeton NJ) in 500 mL dextran in the peritoneal cavity at the time of closure as previously published by our group [24].

Operative technique consisted of a Pfannenstiel incision, pitressin (20U in 60 mL normal saline) was slowly injected into the center of each myoma using an 18-guage spinal needle. Uterine incisions were made using electrocautery in a transverse direction to avoid the arcuate vessels and were carried through the serosa, myometrium and pseudocapsule using needlepoint electrode. The myoma was grasped with a tenaculum and the overlying myometrium and pseudocapsule were bluntly dissected off the myoma. Care was taken not to enter the uterine cavity unless the fibroid was classified as International Federation of Gynecology and Obstetrics (FIGO) type 2, 3 or 2–5, in which case removing the entire fibroid was prioritized. The defect was closed in multiple layers using running 0-polyglactin (Vicryl, Johnson&Johnson) suture.

### 2.3 Data analysis

Data previously collected in an Excel Spreadsheet was analyzed using Prism (Graphpad, San Diego, CA) and Wizard Pro for Mac. Descriptive statistics were used to characterize the dataset. Pearson correlation was performed to test for correlation, Student t-test was used to compare groups assuming unequal variance (F-test) and two tails. Pearson Chi-squared test was performed on categorical data. Significance was set with p<0.05.

### 2.4 Ethics statement

This study was approved by the institutional review board (IRB) at St. Vincent’s Catholic Medical Centers Manhattan, (IRB# 010419M1X), and involved a retrospective chart review. All data was de-identified and informed consent was waved by the IRB for this retrospective chart review. All procedures performed in studies involving human participants were in accordance with the ethical standards of the institutional and national research committee and with the 1964 Declaration of Helsinki and its later amendments or comparable ethical standards.

## 3. Results

### 3.1. Sample population

During the study period, 592 primary open myomectomies for large symptomatic fibroid uterus were performed and these patients were followed. Out of this group, 72 uterine surgeries were performed in patients who had previously undergone myomectomy. Mean age at reoperation was 44.1 ± 0.6 years (range 33–53) and racial composition was as follows: Non-Hispanic White: 29.0%, African American: 52.2%, Hispanic: 10.1%, Other: 8.7%. The mean time from initial surgery to second surgery was 7.9 ±0.3 years (range: 5–15 years).

### 3.2. Rates of reoperation

**Fig 1:**
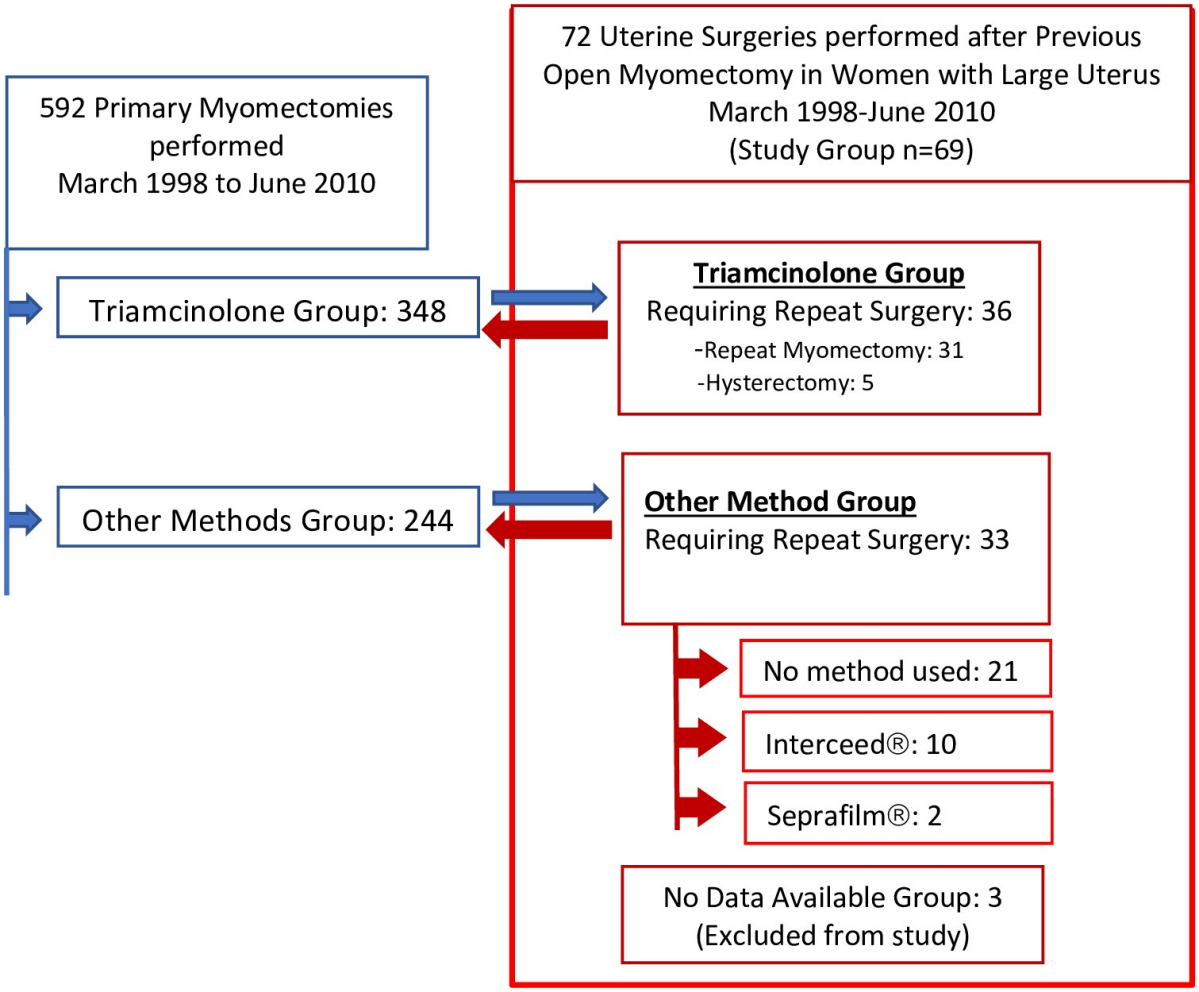
Summary of study cohort. Our cohort consisted of 72 patients who underwent reoperation for a recurrence of symptoms attributable to fibroid uterus after having undergone a primary abdominal myomectomy for large myomas. Data was available on n = 69 patients.

Five hundred and ninety-two patients were followed and seventy-two patients (12%) from this group chose to undergo uterine surgery for their symptoms at a later date. Data was available for n = 69 patients (Fig 1).

### 3.3. Indication for repeat uterine surgery

Indications for uterine surgery after previous myomectomy in our sample consisted of: abnormal uterine bleeding secondary to fibroids (37.7%), dyspareunia/dysmenorrhea refractory to medical management (24.6%), pelvic pressure (13.0%), infertility (17.4%), as well as other symptoms, such as urinary issues, attributed to fibroid uterus (7.8%). Although patients were extensively counselled on alternatives, including hysterectomy, to repeat open myomectomy, patients cited the following reasons for uterine conserving surgery: desire to maintain fertility (40%), belief that having a uterus/cervix enhances sexual pleasure (50%), belief that having a cervix prevents prolapse (10%).

### 3.4. Time to reoperation

**Fig 2:**
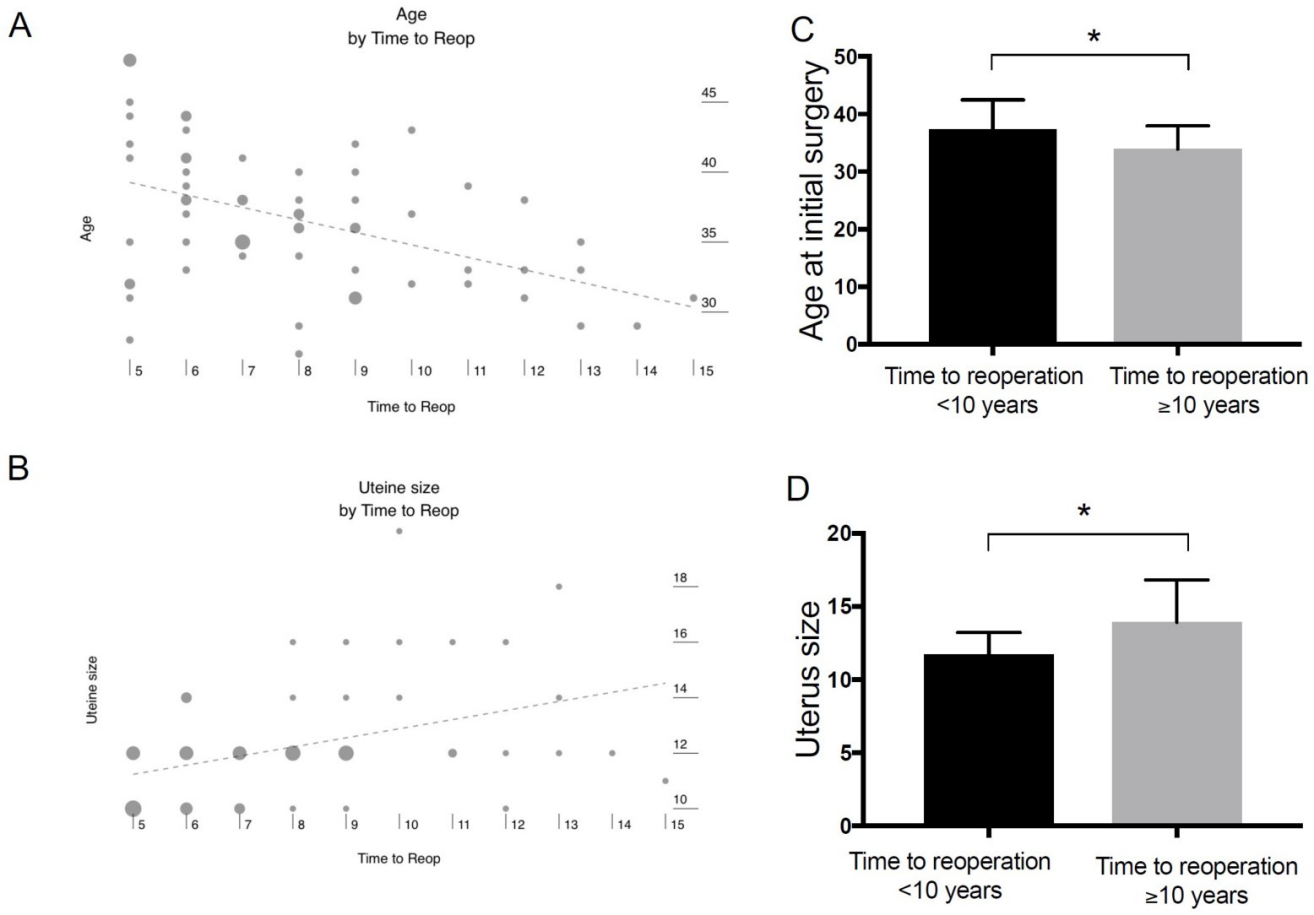
Timing of reoperation. Time between first surgery and patient presenting for repeat uterine surgery was correlated with (**A)** age at initial surgery (correlation = -0.46, R2 = 0.21, p<0.001) and (**B)** uterine size at reoperation (correlation = 0.41, R2 = 0.16, p<0.001). Reoperation a decade or more after initial surgery was significantly correlated with (**C)** younger age at the initial surgery(* p<0.031) and (**D)** larger uterus (* p<0.008).

The time between the first surgery and the patient presenting for her second uterine surgery was, in this study, not correlated with race, the number of fibroids or the aggregate weight of fibroids removed during the initial surgery nor with the operative time or EBL at initial procedure, unlike reported in other studies [25]. Time to reoperation was, however, significantly correlated with both age at initial surgery (correlation = -0.40, R2 = 0.16, p<0.001) and uterine size at reoperation (correlation = 0.42, R2 = 0.18, p<0.001) (Fig 2A and 2B). Published non-modifiable risk factors for recurrence include uterine size, premenopausal status and age [26], which our results confirmed. Reoperation a decade or more after the initial surgery was significantly correlated with younger age at the initial surgery (p = 0.031) and a larger uterus (p<0.008) (Fig 2C and 2D).

### 3.5 Uterine size at reoperation

When presenting for repeat surgery, mean uterine size was 12.2 ±0.2 weeks (range: 10–20 weeks) consistent with ultrasound. This was a significantly (p<0.001) smaller than when they presented for their initial surgery, which had a mean uterine size of 20.8 ±0.5 weeks (range: 12–30 weeks). Presentation at recurrence of fibroids was highly variable and included bleeding, pelvic pressure and pain with a considerable number of patients complaining of a combination of these symptoms. Indication for pain was associated with older ages, while infertility was associated with younger ages (mean age of 46.7 vs 38.9 respectively, p<0.001). Uterine size at reoperation was found to be independent of race.

### 3.6. Use of adhesion-reducing methods at the time of myomectomy

The Triamcinolone group (n = 348) was compared to the “other method group” (n = 244) and no significant differences were found regarding age, number of fibroids removed, aggregate weight of fibroids, blood loss at surgery and initial uterine size. Data was analyzed to determine if the use of intraperitoneal triamcinolone with dextran (n = 36) or adhesion barriers such as Seprafilm (n = 2) or Interceed (n = 10) at the time of the initial myomectomy influenced timing of fibroid recurrence over untreated patients (n = 21). Neither steroids nor adhesion barriers significantly delayed timing for reoperation.

Data was analyzed to determine if the use of triamcinolone would alter the rate of patients with symptoms necessitating repeat uterine surgery. Data from a previous study on postoperative adhesions consisted of a group of 348 patients in the arm receiving triamcinolone. These patients were followed longitudinally and 36 patients (10%) underwent subsequent uterine surgery during the study period. For comparison, 33 women, out of the 244 primary myomectomies that were performed using either no adhesion barriers or commercially available products as described previously, underwent repeat surgery with all but two choosing repeat myomectomy. This difference (10% vs 13%) was statistically significant (p<0.001).

## 4. Discussion

Uterine fibroids are a common condition and, in the United States, incur an estimated annual direct cost of $4.1 to $9.4 billion [27]. Despite counseling on the risks of recurrence, many women prefer to opt for uterine sparing procedures citing concerns regarding fertility and sexual pleasure. Recurrence of the symptoms of fibroids is associated with increased costs, hospital admissions, transfusions, and pain for the patient.

In this study, we have examined the recurrence rates of uterine leiomyomas leading to reoperation after open myomectomy on a large fibroid uterus. Our rate of reoperation of about 12% seems lower than the average, with some studies citing recurrence rates of 23%, following open myomectomy, and slightly higher rates following laparoscopic procedures [21, 28]. One well-constructed study which followed patients after myomectomy [17] determined that about 21% of women had a second surgery, 75% of which were hysterectomies. Our results were significantly lower. It is unclear if this is because of the use of a more meticulous open technique where even small palpable fibroids are removed before they have time to grow larger while in comparison to that study, the type of initial myomectomy was performed through a combination of approaches. In minimally invasive myomectomies, where the surgeon can’t palpate small fibroids, preoperative investigations (ultrasound and magnetic resonance imaging) and intraoperative laparoscopic ultrasound [29] are important to identify and remove small “occult” fibroids, thus preventing them from growing to a size that may in some period of time later cause symptoms. Additionally, such modalities can help identify if other pathologies, such as adenomyosis, are present (potentially contributing to symptoms) and ensure that the patient receives appropriate management. Our approach in counselling patients with recurrence of symptoms was individualized and also emphasized watchful waiting or medical management, particularly in older women. As a center for myomectomy, possible bias in counselling may have resulted in a greater ratio of repeat myomectomy over hysterectomy. Ultimately, in a shared decision-making approach, the patient made the choice of treatment, weighing risks, benefits and alternatives as it applied to her.

Our study found no correlation between the time to reoperation and race, number of fibroids, or aggregate weight. This contrasts with previous studies that established a correlation between reoperation rates and number of fibroids: recurrence rates with a single fibroid was 9.5% vs 24.9% with multiple myomectomy [18, 26]. Since we have shown that age is tightly correlated with timing of recurrence, this result may be due to the difference in average age of women in their study (29.8 ± 3.8) compared to our study (36.1 ± 0.6 years old). Additionally, our myomectomies were performed in larger uteri via laparotomy, which may also influence recurrence rates as one of the limitations of minimally invasive surgery is the lack of tactile sensation, making it difficult to detect smaller fibroids embedded in the myometrium.

However, we did find a significant correlation between time to reoperation and both age at initial diagnosis and uterine size at reoperation, which is consistent with the data sets of other studies [21, 25, 26]. This suggests that longer time spans allow fibroids to grow more; however, this may be a bias introduced by patients, who presented for surgical management. Patients, who were older at the time of initial myomectomy, who returned with symptomatic fibroid uterus, may have been counselled on watchful waiting until menopause or alternative (non-surgical) treatment methods.

Patients presented again primarily with complaints of abnormal uterine bleeding and pain (dyspareunia/dysmenorrhea) and surgery performed for indication of “pain” were significantly associated with older ages. Younger patients, however, presented again for infertility and were counselled on pregnancy outcomes following repeat myomectomy [30]. The subsequent presentation was associated with a smaller burden of fibroid tumor than at initial presentation, suggesting that either patients tended to recognize these symptoms earlier or that emergence of symptoms occurred at a smaller uterine size. Thus, patients presented with uterus sizes significantly smaller than when they presented for their original myomectomy and may opt to repeat an approach which previously worked for them instead of delaying care until symptoms worsen significantly.

One of the weaknesses of this study, is that it consists of a retrospective analysis of women, who elected to undergo surgical management. This self-selected group of patients may not reflect entirely the cohort of patients, who have a recurrence of symptoms but choose an alternative approach. For instance, this study did not include women with ultrasound findings of recurrent fibroids, who are asymptomatic, women, who are symptomatic but choose watchful waiting or a conservative approach or women, who choose other surgical approaches such as embolization. Thus, in this study, the recurrence rates are reflective of patients with recurrence of symptoms, who elected to have a repeat surgery. Another weakness involves the use of bimanual exam to determine, in conjunction with ultrasound, clinical uterine size. This type of exam yields important pre-surgical information (such as mobility of the uterus), laxity in the tissues, presence of other gynecological pathology and helps with surgical planning, particularly when fibroids are close to the cervix. Additionally, bimanual sizing is highly reproducible among gynecologists.

In this study, the use of intraperitoneal triamcinolone and dextran as an approach to limit postoperative adhesion formation [24] was associated with significantly lower rates of recurrence requiring repeat surgery (10% vs 13%, p<0.001) when compared to not using anti-adhesion measures or using barrier methods as well as to recurrence rates from other published studies cited herewith. It is unclear if this difference is from steroid administration or surgical technique. Analysis of the time to recurrence as a function of patients having received either no treatment or steroids or the adhesion barriers Seprafilm or Interceed found no significant correlations. Further analysis may be necessary as “time to recurrence” (and presenting for surgery) and “recurrence rates” are related but distinct quantities.

The cellular origin of uterine fibroids remains unknown [31] but genetic studies support that they are monoclonal tumors [32]. A mutation in MED12 or HMGA2 [33] in myometrial stem cells [34] may be the precipitating event. Under estrogen and progesterone stimulation, mature myometrial cells secrete paracrine factors, such as WNT ligands, which activate the β-catenin-T-cell transcription factor pathway through the Frizzled receptor [35] and inducing TGF-B production [36]. In mutated cells this triggers proliferation through the smad pathway. Thus, after an open myomectomy, it is possible that some mutated fibroid stem cells, which were too small to be seen or palpated, were left behind. With time, as well as through de-novo transformation of myometrial cells, these fibroids can proliferate in an exponential fashion, and become symptomatic.

At the cellular level, we hypothesize that triamcinolone may abate fibroid recurrence by downregulating fibroid growth factors, principally TGF-β3 [37, 38], which is one of the principal drivers of uterine smooth muscle and extracellular matrix proliferation. Our group has shown that intraperitoneal steroids decrease adhesion formation through a reduction in TGF-β at hypoxia levels typically found in the peritoneum. As a next step, blocking the TGF-β pathway through an ALK5 blockade, such as by using SB525334/SB 505124 [39], may decrease activation of smad 2/3 and the transcription factors involved in smooth muscle proliferation.

## 5. Conclusion

This study followed a group of women who chose to undergo repeat surgery for recurrence of symptomatic fibroid uterus after an initial open myomectomy for large size myomatous uterus. Time to reoperation was correlated to age and to fibroid size but not to race or number of fibroids removed, which may be an important part of patient counselling, particularly in younger women. Further research is necessary to assess how gynecologists can prevent the recurrence of fibroids in patients who undergo uterus sparing procedures. Novel interventions, such as intraperitoneal triamcinolone at the time of initial surgery, may potentially play a role in reducing reoperation rates.

## Supporting information

S1 File (XLSX)
